# The association of incident abdominal obesity with diet and unfavorable lifestyle factors in Korean adults: A prospective cohort study

**DOI:** 10.1097/MD.0000000000046422

**Published:** 2026-01-02

**Authors:** Jieun Kim, Kyoungsik Jeong, Siwoo Lee, Younghwa Baek

**Affiliations:** a Division of Korean Medicine Data, Korea Institute of Oriental Medicine, Daejeon, South Korea.

**Keywords:** abdominal obesity, diet, lifestyle, metabolic syndrome, prospective cohort

## Abstract

Unhealthy lifestyles are strongly associated with the development of cardiometabolic diseases and the risk of all-cause mortality. We hypothesized that unhealthy lifestyles including poor diet contribute to the development of cardiometabolic diseases. This study aimed to investigate the incidence of metabolic syndrome (MetS) and its clustering components in relation to diet and lifestyle risk factors in Korean population. At baseline, all participants (n = 2000) completed a general health-related questionnaire, provided blood samples, and underwent health examinations. Lifestyle factors were investigated including smoking, alcohol consumption, physical activity, sleep, dietary quality, and weight status. Cox proportional hazard regression was used to estimate the hazard ratio (HR) and 95% confidence intervals (CIs) of primary endpoints: MetS events and their clustering components. A total of 1697 participants (mean age: 43.8 ± 0.2 years) were included, of whom 72.4% (n = 1228) were women. Compared to the healthy group, unhealthy participants had a significant HR value of abdominal obesity for lifestyle factors (HRs: 1.937, 95% CI: 1.335–2.810). No significant differences were observed between MetS and other clustering components. After adjusting for covariates, the lowest quintile of vitamin C (HRs: 1.553, 95% CI: 1.153–2.091), fiber (HRs: 1.409, 95% CI: 1.035–1.919) and vegetable intake (HRs: 1.300, 95% CI: 1.008–1.678) were significantly associated with abdominal obesity incidence compared with the highest quintile. Adherence to healthy lifestyle factors and dietary factors, such as high intakes of vitamin C and vegetables, were negatively associated with the incidence of abdominal obesity among the MetS components in Korean adults.

## 1. Introduction

Metabolic syndrome (MetS) and its components, including abdominal obesity, hypertriglyceridemia, decreased high-density lipoprotein cholesterol (HDL-C) level, high blood pressure (BP), and/or impaired glucose tolerance, are major public health challenges.^[1]^ Globally, obesity and MetS have increased rapidly and become highly prevalent over the past 3 decades.^[2,3]^ Approximately one in 3 adults in Korea struggles with MetS.^[4]^ Recently, an association between MetS development and lifetime risk of cardiovascular disease (CVD) has been reported.^[5]^

Metabolically, individuals with adverse metabolic profiles are at high risk of diabetes mellitus, CVD, and mortality.^[6,7]^ Abdominal obesity is a rising threat to the development of MetS in both obese and non-obese people.^[8]^ The global prevalence of abdominal obesity has shown an increasing trend in young adults since the 1990s from 31.3% (1985–1999) to 48.3% (2010–2014) in more than 280 population-based studies.^[9]^ Similarly, an upward trend in the prevalence of obesity and abdominal obesity was reported in young Korean men^[10]^ and a high risk of major risk factors for CVD in abdominally obese individuals without obesity among 315,982 Koreans.^[11]^

Unhealthy lifestyles (e.g., smoking, alcohol consumption, physical inactivity, and adherence to an unhealthy diet) are strongly associated with the development of cardiometabolic diseases and the risk of all-cause mortality in multinational prospective cohort studies.^[12,13]^ In particular, an unhealthy diet has contributed to 11 million deaths and 255 DALYs (disability-adjusted life-year) in 195 countries.^[14]^ A recent Korean population-based study reported that several poor lifestyle factors tended to increase the risk of MetS in Korean adults.^[15]^ In contrast, the combined impact of a healthy diet and lifestyle factors was found to be the most protective against cardiometabolic diseases, cancer, and all-cause mortality.^[16]^ Nevertheless, the beneficial effects of a combined lifestyle on an individual’s lifelong health have been explored in only a few long-term observational studies.^[17–19]^ We hypothesized that unhealthy lifestyles including diet negatively affects to the development of cardiometabolic diseases. Therefore, this study aimed to identify the incidence of MetS and its clustering components according to dietary and lifestyle risk factors in Korean population.

## 2. Materials and methods

### 2.1. Data source and study participants

The Korean Medicine Daejeon Citizen Cohort (KDCC) is the first prospective ongoing observational cohort study that considers traditional Korean medicine (KM) in assessing risk factors associated with cardiometabolic diseases in South Korean adults^[20]^ (Trial registration number: KCT0004297). Eligible participants aged between 30 to 55 years, free of cancer and CVD such as myocardial infarction, angina, or stroke were recruited from 2017 to 2019 at baseline (n = 2000) and from 2020 to 2021 at follow-up (n = 1441). Individuals with clinically diagnosed CVD including myocardial infarction, angina, or stroke, and those with a history of cancer were excluded to minimize potential confounding effects of disease severity, treatment, and altered dietary behaviors.^[20]^ At baseline, all participants completed a general health-related questionnaire, provided blood samples, and underwent health examinations. After excluding participants diagnosed with MetS at baseline, without information on Mets components (n = 287), or with implausible energy intake (<500 or ≥5000 kcal/day; n = 16) based on previous nutritional epidemiologic studies,^[21–23]^, a total of 1697 participants were enrolled.

All data were handled using the web-based Korean Medicine Data Center (KDC) electronic data capture system of the Korean Institute of Oriental Medicine.

### 2.2. Ethical approval

Ethical approval was obtained after participants provided written informed consent. This study was approved by the Ethics Committee of the Korean Institute of Oriental Medicine (IRB No. I-1703/002-002, DJDSKH-17-BM-12).

### 2.3. Lifestyle factors

We defined the group rationale of a combination of lifestyle factors by adopting the previous large and diverse races population-based studies^[13,18,24–26]^ to evaluate the health status of the present study participants. Lifestyle factors of the participants were collected by using self-reported questionnaires on smoking (nonsmoker; having smoked <100 cigarettes or ≥100 cigarettes in their lifetime but not smoking currently, vs smokers; having smoked ≥100 cigarettes in their lifetime), alcohol consumption (non-heavy; ≤7 drinks/week for women or ≤14 drinks/week for men vs heavy; >7 drinks/week for women or >14 drinks/week for men), physical activity (total MET (metabolic equivalent) minutes per week: insufficient (<600 MET-min/week), sufficient (≥600 MET-min/week)), sleep quality (good: Pittsburgh Sleep Quality Index (PSQI) level ≤5 or poor: PSQI > 5), dietary quality (low (Q1 and Q2) or high (Q3 and Q4) by total Korean Healthy Eating Index score, and weight status (using body mass index (BMI)). The BMI was calculated as weight in kilograms divided by height in meters squared (kg/m^2^). According to the BMI weight status categories, participants were classified as obese when BMI was ≥25kg/m^2^ and as non-obese when <25kg/m^2^. Binary outcomes (healthy factor = 1, unhealthy factor = 0) were scored for each item. None of the participants had 6 factors in the present study. Therefore, total lifestyle scores were created based on the 6 lifestyle factors (0–5 factors) and classified into 3 groups: unhealthy (0–1 factors), moderate (2–3 factors), and healthy (4–5 factors).

### 2.4. Definition of MetS and its components

MetS is defined as the cooccurrence of more than 3 of the following 5 components^[27]^: abdominal obesity based on waist circumference (WC), with cutoff points specific to South Koreans (WC ≥ 90 cm in men and ≥85 cm in women); reduced HDL-C <40 mg/dL in men and <50 mg/dL in women or drug treatment for this lipid abnormality; increased triglycerides (TG) to more than equal to 150 mg/dL or specific treatment for this lipid abnormality; elevated BP with a systolic BP (SBP) more than equal to 130 mm Hg, a diastolic BP (DBP) more than equal to 85 mm Hg, or treatment of previously diagnosed hypertension; increased fasting blood glucose (FBG) more than equal to 100 mg/dL or antidiabetic drug treatment for diabetes mellitus.

### 2.5. Dietary assessment

The participants’ dietary intake was assessed using a validated short-form food frequency questionnaire (SF-FFQ) (20), which contained 34 food items with serving sizes. In the SF-FFQ, participants were asked to report their usual intake frequency (with 9 stages: almost null, 1 time/month, 2–3 times/month, 1–2 times/week, 3–4 times/week, 5–6 times/week, 1 time/day, 2 times/day, or 3 times/day) and portion size (three or 4 specified portion sizes) of each food item in the preceding year.

The daily intake for each food item was converted to daily food quantity and type of nutrient, which was then calculated as the total energy (kcal/day), macronutrient (carbohydrate, fat, and protein) and micronutrient (fiber, vitamin A, vitamin D, vitamin E, vitamin B1, vitamin B2, vitamin C, vitamin K, folate, sodium, calcium, phosphorus, potassium, magnesium, iron, and zinc) (g/day), and food group (white rice, mixed rice, noodles and bread, potatoes and sweet potatoes, beans and tofu, fish, beef and pork, poultry and eggs, fruits and vegetables, milk and yogurt, hamburger and pizza) intakes using a computer-aided nutritional analysis program (CAN Pro, Version 5.0, The Korean Nutrition Society, 2015). This program was based on the recommended nutritional intake from the Dietary Reference Intake for Koreans (Korean Nutrients Society, 2020).

### 2.6. Follow-up and outcome variable

**Figure 1.**
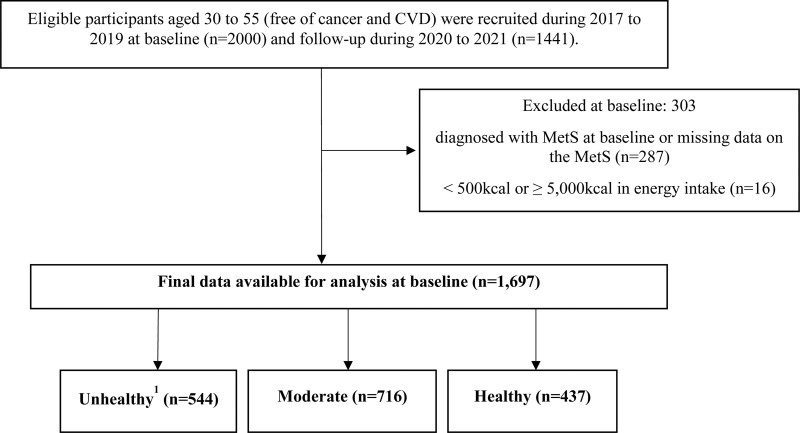
Flow diagram illustrating the selection of subjects for analysis. Note: Total lifestyle scores were created based on the 6 lifestyle factors (range: 0–6 factors); smoking (nonsmoker or smokers), alcohol consumption (light or heavy), physical activity (total MET [metabolic equivalent] minutes per week: insufficient [<600 MET-min/wk]); sufficient (≥600 MET-min/wk)), sleep quality (good: Pittsburgh Sleep Quality Index (PSQI) level ≤ 5 or poor: PSQI > 5), dietary quality (lower (Q1 and Q2) or higher (Q3 and Q4) by total KHEI (Korean Healthy Eating Index) score) and body mass index (BMI) and classified into 3 groups: unhealthy, 0–1 factors; moderate, 2–3 factors; and healthy, 4–5 factors.

The primary outcome variable was the incidence of MetS and its components in relation to lifestyle factors. The secondary outcome was the incidence of MetS and its components in relation to dietary factors. Person-years were calculated using the estimated date of onset of MetS and its components. Follow-up time was defined as the time from the cohort entry to the first diagnosis of MetS and its components or end of follow-up, whichever came first. Hazard ratios (HRs) and 95% confidence intervals (CI) of MetS risk and those with more than 1 year of follow-up were included in this study (median follow-up: 2.2 years) (Fig. 1).

### 2.7. Statistical analyses

**Figure 2.**
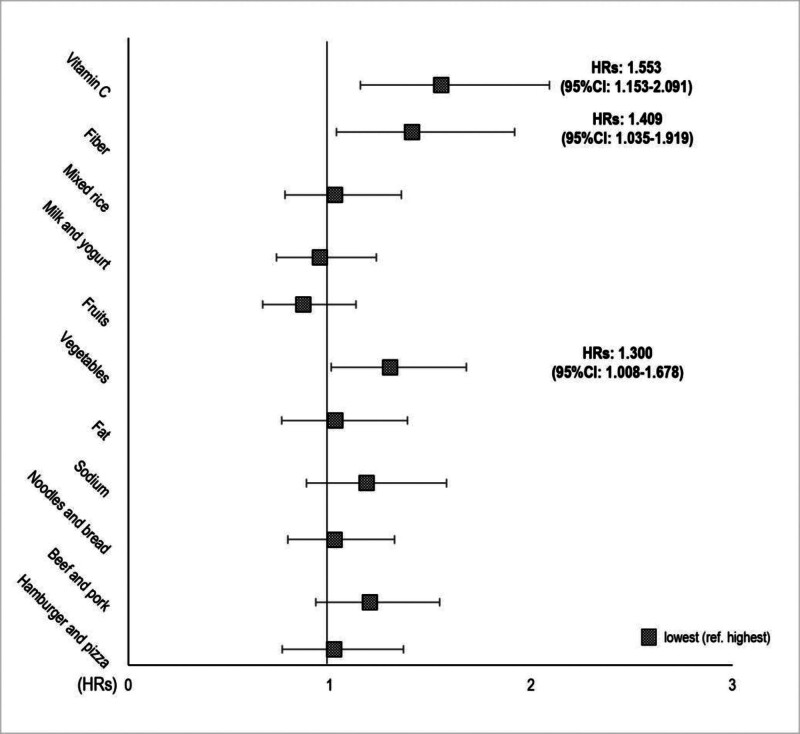
Hazard ratios (HRs) and 95% confidence intervals (CIs) for abdominal obesity according to dietary factors. Cox proportional hazards regression analysis was performed after adjusting for age, sex, BMI, education level, household income, energy intake (kcal), and lifestyle status. BMI = body mass index.

Frequencies and percentages were used as categorical variables in descriptive analyses. The chi-square (χ^2^) test was used to compare the general and health-related characteristics (age, sex, smoking, alcohol consumption, physical activity, PSQI score, Korean Healthy Eating Index score, and weight status) in the descriptive analysis (Table 1). Normality was tested for all continuous variables using the Shapiro–Wilk test. All data on continuous variables related to cardiometabolic risk factors (WC, SBP, DBP, FBG, HDL-C, and TG) (Table 1), and energy (kcal), nutrient intake, and food groups are presented as means and standard errors (Table 2). Survival curves were plotted for total patients, using the Kaplan–Meier method and compared with the log-rank test (Supplementary File S1, Supplemental Digital Content, https://links.lww.com/MD/Q847). The assumption of proportional hazards for each variable were tested in the model as a function of follow-up time. HRs and 95% CIs were calculated using multivariate-adjusted with adjustments for age, gender, BMI, education level, household income and energy intake (kcal). Cox proportional hazard regression (Table 3 and Fig. 2). All analyses were performed using SAS version 9.4 (SAS Institute Inc., Cary). All statistical tests were two-tailed, and *P*-values <.05 indicated statistical significance.

**Table 1:** General characteristics, lifestyle factors and cardiometabolic risk factors according to individual lifestyle scores (unhealthy, 0–1 factors; moderate, 2–3 factors; and healthy, 4–5 factors) based on the 6 lifestyle factors at baseline.

General characteristics	All (n = 1697)	Unhealthy (n = 544)	Moderate (n = 716)	Healthy (n = 437)	*P*-value
Age (yr), (mean ± SE)					
30–44 (n, %)	894 (52.7)	285 (52.4)	402 (56.1)	207 (47.4)	**.015**
45–55	803 (47.3)	259 (47.6)	314 (43.9)	230 (52.6)	
Gender (%)					
Men	469 (27.6)	233 (42.8)	160 (22.4)	76 (17.4)	**<.0001**
Women	1228 (72.4)	311 (57.2)	556 (77.6)	361 (82.6)	
Education level					
High school lower levels	597 (35.2)	189 (34.7)	260 (36.3)	148 (33.9)	.677
College and higher levels	1100 (64.8)	355 (65.3)	456 (63.7)	289 (66.1)	
Household income*					
Low	302 (17.8)	105 (19.3)	142 (19.8)	55 (12.6)	**.026**
Middle	1225 (72.2)	387 (71.1)	504 (70.4)	334 (76.4)	
High	170 (10.0)	52 (9.6)	70 (9.8)	48 (11.0)	
Lifestyle factors					
Smoking status† (%)					
Nonsmoker	1511 (89.0)	458 (84.2)	634 (88.5)	419 (95.9)	**<.0001**
Smoker	186 (11.0)	86 (15.8)	82 (11.5)	18 (4.1)	
Alcohol consumption‡ (%)					
Non-heavy	1048 (61.8)	298 (54.8)	428 (59.8)	322 (73.7)	**<.0001**
Heavy	649 (38.2)	246 (45.2)	288 (40.2)	115 (26.3)	
Physical activity§ (%)					
Insufficient	523 (30.8)	167 (30.7)	219 (30.6)	137 (31.3)	.434
Sufficient	1174 (69.2)	377 (69.3)	497 (69.4)	300 (68.7)	
PSQI‖					
Poor	550 (32.4)	178 (32.7)	371 (51.8)	1 (0.2)	**<.0001**
Good	1147 (67.6)	366 (67.3)	345 (48.2)	436 (99.8)	
KHEI¶					
Poor	824 (48.6)	287 (52.8)	537 (75.0)	0 (0.0)	**<.0001**
Good	872 (51.4)	256 (47.2)	179 (25.0)	437 (100.0)	
Weight status (body mass index, kg/m^2^)					
Non-obese	1160 (68.4)	7 (1.3)	716 (100.0)	437 (100.0)	**<.0001**
Obese	537 (31.6)	537 (98.7)	0 (0.0)	0 (0.0)	
Cardiometabolic risk factors#					
WC (cm)		91.1 ± 0.3^a^	81.1 ± 0.3^b^	80.9 ± 0.4^b^	**<.0001**
Systolic BP (mm Hg)		123.3 ± 0.7^a^	117.1 ± 0.6^b^	116.6 ± 0.8^b^	**<.0001**
Diastolic BP (mm Hg)		77.8 ± 0.5^a^	73.4 ± 0.5^b^	72.4 ± 0.6^b^	**<.0001**
FBG (mg/dL)		89.6 ± 0.7^a^	87.3 ± 0.6^b^	86.4 ± 0.8^b^	**<.0001**
HDL-c (mg/dL)		52.9 ± 0.7^b^	58.0 ± 0.6^a^	57.8 ± 0.8^a^	**<.0001**
TG (mg/dL)		140.4 ± 3.9^a^	126.4 ± 3.7^b^	122.5 ± 4.0^b^	**.004**
Follow up years	2.2				

*P* values were obtained from Rao–Scott χ^2^ tests for categorical variables and Bonferroni multiple comparison of one-way analysis of variance and analysis of covariance (ANCOVA). Bold values indicate results that are statistically significant (*P* < .05).

a, b, and c indicate statistically significant ordered differences among groups (*P* < .05), based on ANCOVA followed by Bonferroni multiple comparison test.

ANCOVA = way analysis of variance and analysis of covariance, BP = blood pressure, FBG = fasting blood glucose, HDL-c = high density lipoprotein cholesterol, KHEI = Korean Healthy Eating Index, MET = metabolic equivalent of task, PSQI = Pittsburgh Sleep Quality Index, TG = triglyceride, WC = waist circumference.

* Monthly household income was divided into 3 groups: low (<3,000,000 won), middle (3,000,000–6,999,000 won), and high (≥7,000,000 won).

† Smoking status was categorized as smoker (if they had smoked at least 100 cigarettes in their lifetime) or non-smoker.

‡ Alcohol consumption was evaluated as non-heavy or heavy drinkers (defined as alcohol intake ≥ 5 glasses for male and ≥ 4 glasses for female subjects on one occasion ≥ once a week).

§ Physical activity: total MET minutes per week: insufficient (<600 MET-min/wk); sufficient (≥600 MET-min/wk).

‖ Global sleep score (GSS) was classified into 2 different level; good: GSS ≤ 5 or poor: 5 < GSS by Pittsburgh Sleep Quality Index (PSQI) level.

¶ Total KHEI (Korean Healthy Eating Index) score was classified into 2 different level; lower (Q1 and Q2) or higher (Q3 and Q4).

# Least-square means ± SE adjusted for age and sex.

**Table 2:** Group differences in dietary factors according to the individual lifestyle scores (unhealthy, 0–1 factors; moderate, 2–3 factors; and healthy, 4–5 factors) based on the 6 lifestyle factors at baseline.

General characteristics	Unhealthy (n = 544)	Moderate (n = 716)	Healthy (n = 437)	*P*-value
Energy (kcal/d)*				
Men	2193.0 ± 43.8^b^	2248.3 ± 52.8^b^	2489.9 ± 77.1^a^	**.004**
Women	2097.6 ± 38.7^a^	1938.2 ± 29.0^b^	2215.7 ± 35.9^a^	**<.0001**
Nutrients† (per 1000 kcal/d)				
Carbohydrates, g	148.5 ± 0.8^b^	147.5 ± 0.7^b^	155.6 ± 0.9^a^	**<.0001**
Fat, g	24.7 ± 0.2^a^	25.0 ± 0.2^a^	23.2 ± 0.3^b^	**<.0001**
Protein, g	33.4 ± 0.2^ab^	33.4 ± 0.2^a^	32.8 ± 0.2^b^	**.023**
C:F: P (%)	59.4:22.2:13.4	59.0:22.5:13.4	62.2:20.8:13.1	**<.0001**
Fiber, g	9.9 ± 0.1	9.8 ± 0.1	10.1 ± 0.1	.154
Vitamin A, µg RAE	149.1 ± 1.9	147.7 ± 1.8	145.4 ± 2.3	.437
Vitamin D, µg	2.2 ± 0.0	2.1 ± 0.0	2.1 ± 0.0	.122
Vitamin E, mg	7.2 ± 0.1^ab^	7.3 ± 0.1^a^	6.9 ± 0.1^b^	**.007**
Vitamin B1, mg	0.8 ± 0.0	0.8 ± 0.0	0.8 ± 0.0	.231
Vitamin B2, mg	0.7 ± 0.0	0.7 ± 0.0	0.7 ± 0.0	.121
Vitamin C, mg	33.3 ± 0.6^ab^	31.6 ± 0.6^b^	35.1 ± 0.8^a^	**.001**
Vitamin K, mg	39.0 ± 0.8	40.2 ± 0.7	38.2 ± 1.0	.200
Folate, µg	200.4 ± 1.8	197.6 ± 1.7	201.9 ± 2.1	.222
Sodium, mg	1455.1 ± 14.5^a^	1478.3 ± 13.7^a^	1363.9 ± 17.5^b^	**<.0001**
Calcium, mg	212.2 ± 2.8	206.1 ± 2.6	214.9 ± 3.3	.060
Phosphorus, mg	523.7 ± 3.1	517.1 ± 2.9	527.1 ± 3.7	.057
Potassium, mg	1142.4 ± 9.4	1133.5 ± 8.9	1151.4 ± 11.3	.408
Magnesium, mg	46.1 ± 0.5	45.7 ± 0.5	46.5 ± 0.6	.476
Iron, mg	6.1 ± 0.0	6.0 ± 0.0	6.1 ± 0.0	.582
Zinc, mg	4.9 ± 0.0^b^	4.9 ± 0.0^b^	5.0 ± 0.0^a^	**.000**
Food groups (g per 1000 kcal/d)‡				
White rice	91.2 ± 1.7^ab^	90.7 ± 1.6^b^	97.2 ± 2.1^a^	**.022**
Mixed rice	33.4 ± 1.5^ab^	30.7 ± 1.4^b^	38.3 ± 1.7^a^	**<.0001**
Noodles and bread	48.1 ± 1.6^b^	54.1 ± 1.5^a^	37.1 ± 1.9^c^	**<.0001**
Potatoes and sweet potatoes	21.0 ± 1.3	21.9 ± 1.3	19.7 ± 1.6	.502
Beans and tofu	12.8 ± 0.5	12.6 ± 0.5	12.4 ± 0.6	.907
Fish	15.1 ± 0.3^ab^	15.8 ± 0.3^a^	14.1 ± 0.4^b^	**.002**
Beef and pork	47.0 ± 1.6^ab^	51.8 ± 1.6^a^	41.3 ± 2.0^b^	**<.0001**
Poultry and eggs	32.9 ± 1.0	30.6 ± 1.0	30.5 ± 1.2	.194
Fruits	39.2 ± 1.9^b^	35.8 ± 1.8^b^	52.6 ± 2.3^a^	**<.0001**
Vegetables	17.7 ± 0.5	17.3 ± 0.5	17.6 ± 0.6	.804
Milk and yogurt	48.6 ± 2.3^ab^	44.9 ± 2.2^b^	55.8 ± 2.8^a^	**.005**
Hamburger and pizza	42.4 ± 2.5^a^	45.7 ± 2.4^a^	31.9 ± 3.1^b^	**.000**

Bold values indicate results that are statistically significant (*P* < .05).

a, b, and c indicate statistically significant ordered differences among groups (*P* < .05), based on ANCOVA followed by Bonferroni multiple comparison test.

* Adjusted age only.

† Least-square means ± SE adjusted for age, sex and energy intake (kcal).

‡ Food groups were surveyed using the short-form of the food frequency questionnaire (FFQ) and categorized into twelve food groups.

**Table 3:** HRs and 95% CI for MetS and abdominal obesity according to individual lifestyle scores (unhealthy, 0–1 factors; moderate, 2–3 factors; and healthy, 4–5 factors) based on the 6 lifestyle factors.

	MetS (n = 126)			Abdominal obesity (n = 375)		
Lifestyle factors	Person-years	Incident cases	HRs (95% CI)	Person-years	Incident cases	HRs (95% CI)
Healthy (4–5 factors)	707.9	14	Ref.	707.9	49	Ref.
Moderate (2–3 factors)	1109.9	31	1.324 (0.702–2.496)	1107.3	76	0.979 (0.682–1.405)
Unhealthy (0–1 factors)	826.4	81	1.910 (0.968–3.770)	829.0	250	1.937 (1.335–2.810)

Cox proportional hazard regression was performed after adjusting age, gender, BMI, education level, household income and energy intake (kcal).

BMI = body mass index, CI = confidence interval, HRs = hazard ratios, MetS = metabolic syndrome.

## 3. Results

A total of 1697 participants (mean age 43.8 ± 0.2 years) were included, and 72.4% (n = 1228) of the participants were women. One-third of the participants had an unhealthy lifestyle (n = 544). Individuals in the unhealthy lifestyle group were more likely to be men, smokers, heavy drinkers, and obese (*P* < .0001) than the other 2 groups. Healthy individuals were older, middle-aged, and had better sleep and diet quality (*P* < .0001). No significant differences were observed in educational level or physical activity according to lifestyle status.

Cardiometabolic risk factor statistics showed that significantly higher WC (91.1 ± 0.3cm), SBP (123.3 ± 0.7 mm Hg), DBP (77.8 ± 0.5 mm Hg), FBG (89.6 ± 0.7 mg/dL), TG level (140.4 ± 3.9 mg/dL), and lower HDL-C level (52.9 ± 0.7 mg/dL) were observed in the unhealthy group than in the other 2 groups, respectively (*P* < .0001) (Table 1).

Table 2 shows the daily energy (kcal/day), nutrients (per 1000 kcal/day), and food groups (g per 1000 kcal/day) consumed by participants according to their lifestyle status. A significantly higher energy intake was observed in men and women in the healthy group (*P* < .0001). With regard to nutrients intake, a higher fat (unhealthy: 24.7 ± 0.2 g per 1000 kcal/day, moderate: 25.0 ± 0.2 g per 1000 kcal/day), protein (unhealthy: 33.4 ± 0.2 g per 1000 kcal/day, moderate: 33.4 ± 0.2 g per 1000 kcal/day), and sodium (unhealthy: 1455.1 ± 14.5 mg per 1000 kcal/day, moderate: 1478.3 ± 13.7 mg per 1000 kcal/day) intake were observed in the unhealthy and moderate group, respectively (*P* < .05); whereas, a higher intake of carbohydrates (g), vitamin C (mg), and zinc (mg) were observed in the healthy group (*P* < .01).

Regarding the consumption of food groups, more noodles and bread (unhealthy: 48.1 ± 1.6 g per 1000 kcal/day, moderate: 54.1 ± 1.5 g per 1000 kcal/day), beef and pork (unhealthy: 47.0 ± 1.6 g per 1000 kcal/day, moderate: 51.8 ± 1.6 g per 1000 kcal/day), and hamburger and pizza (unhealthy: 42.4 ± 2.5 g per 1000 kcal/day, moderate: 45.7 ± 2.4 g per 1000 kcal/day) were consumed in the unhealthy and moderate group, respectively (*P* < .05). In contrast, a higher consumption of white rice, mixed rice, fruits, milk and yogurt were consumed in the healthy group (*P* < .05) (Table 2).

The HRs of cardiometabolic risk for the number of lifestyle factors in the participants are presented in Table 3. Compared with the healthy group, a significant HR of abdominal obesity for lifestyle factors was observed in the unhealthy group (HR: 1.937, 95% CI: 1.335–3.770). No significant differences were observed between MetS and other clustering components (elevated BP (n = 253): HR: 1.163, 95% CI: 0.748–1.808; elevated FBG: HR: 1.039, 95% CI: 0.486–2.225; reduced HDL-C or increased triglycerides: HR: 0.872, 9% CI: 0.610–1.247) (data not shown) (Table 3).

The significant HR of abdominal obesity for dietary factors is presented in Figure 2 after adjusting for lifestyle factors and other covariates (age, sex, BMI, education level, household income, and energy intake). The lowest tertile of vegetable and quartile of vitamin C and fiber intake greatly increased the risk of abdominal obesity compared with those in the highest tertile (Fig. 2).

## 4. Discussion

We examined diet and lifestyle factors that were strongly associated with the incidence of MetS and its components in South Korean adults. First, the incidence of abdominal obesity was 1.9 times more in unhealthy participants than in people with healthy lifestyle factors. Second, low intakes of vitamin C, fiber and vegetables were highly linked to the development of abdominal obesity compared with those of their counterparts after adjusting for covariates and lifestyle status.

### 4.1. Lifestyle risk factors in MetS and cardiometabolic diseases

This study demonstrated that unhealthy participants were more likely to be men, smokers, heavy drinkers, and obese. Moreover, cardiometabolic parameters, including higher WC, systolic and diastolic BP, FBG, and TG were observed more in the unhealthy group than in the healthy and moderate groups. Moreover, we observed a higher incidence of abdominal obesity in unhealthy participants than in healthy participants. These findings are consistent with those of other studies^[15,16]^ regarding increased central obesity,^[28]^ higher BMI levels, insulin metabolism, and metabolic syndrome^[29]^ in individuals with low adherence to a healthy lifestyle. Similarly, a greater number of poor lifestyle risk factors such as sleep duration, sedentary time, alcohol use disorders, smoking status, and dietary intake were highly associated with MetS prevalence, abdominal obesity, hypertension, and high TG levels in Korean adults.^[15]^

In contrast, promotion of a healthy lifestyle, such as never smoking, non-daily alcohol intake, medium or high level of physical activity, healthy diet, and BMI, were inversely related to the risk of all-cause and cardiovascular mortality of 0.5 million people in a large population-based study.^[16]^ Another meta-analysis reported that lifestyle modifications, including physical activity and diet, in participants with MetS led to significant decreases in their WC (weighted mean difference (WMD) = −3.12 cm; 95% CI: −4.61, −1.62) and inflammatory indicators (C-reactive protein (WMD: −0.52 mg/mL; 95% CI: −0.72, −0.33) and interleukin-6 (WMD: −0.50; 95% CI: −0.56, −0.45).^[30]^ Similarly, a nationwide lifestyle intervention achieved clinically relevant reductions in WC and BMI and reversal of cardiometabolic risk factors within 3 years.^[31]^

A strong association with WC and cardiovascular risk factors has been observed in several studies.^[32–35]^ Abdominal obesity has been independently associated with hypertension after controlling for BMI in adults with normal BMI and no abdominal obesity in US adults.^[33]^ Furthermore, abdominal obesity also has a greater effect on health and various metabolic complications such as diabetes and hypertension than obesity.^[34,35]^ Therefore, a primary prevention strategy to change poor lifestyle status is required to maintain healthy body weight and WC to prevent MetS and cardiometabolic risk factors.

### 4.2. Effects of dietary sources and patterns on MetS and cardiometabolic diseases

In the present study, our results showed that healthy participants tended to have a prudent diet, whereas a westernized diet with high fat, sodium, wheat, red meat, and fast foods was seen in the unhealthy participants. A slight positive association was consistently reported between central obesity (HR: 1.10, 95% CI: 1.01–1.19), high BP (HR: 1.16, 95% CI: 1.02–1.32), and frequent consumption of fried food.^[36]^ Similarly, a higher adherence to the insulinemic potential of the diet, including margarine, butter, processed meat (sausage), red meat (beef or lamb), French fries, high-energy beverages, and lifestyle, was related to the risk of type-2 diabetes mellitus (T2DM)^[29]^ and increased risk of CVD and CHD.^[37]^ Furthermore, the most hyperinsulinemic or pro-inflammatory dietary patterns were observed in the newly diagnosed type-2 diabetics (Median follow-up: 13.3 years, 11,009 incident cases).^[38]^

Previous observational studies^[39–41]^ have demonstrated the beneficial effects of healthy dietary sources, such as low salt and saturated fatty acid intake, high intake of fruit and vegetables, whole grains, and plant-based prudent diets and patterns.^[42]^ Moreover, several epidemiological studies have shown that dietary antioxidants play a key role in preventing the development of MetS. In a meta-analysis of observational studies, inverse associations were presented between MetS and dietary vitamin C (risk ratio [RR] = 0.93, 95% CI: 0.88–0.97) and circulating vitamin C levels (RR = 0.60, 95% CI: 0.49–0.74; *P* < .001).^[43]^ Another study reported that a two-fold higher consumption of fruits, vegetables, vitamins and fiber was negatively related to the prevalence of MetS in a large national Korean population-based cohort.^[44]^ Similarly, the total intake of vitamins A and C as well as moderate and high fruit intake alleviates MetS in women.^[45]^

In contrast, Westernized and pro-inflammatory diets that are high in processed and animal-based foods, refined grains, and fried foods promote the development of obesity, diabetes, and other cardiometabolic diseases.^[3,46,47]^ High consumption of unhealthy foods rich in saturated fat results in the activation and infiltration of pro-inflammatory immune cells and cytokines, and adipokines, which influence the progression of insulin resistance, T2DM, and obesity-related cardiometabolic diseases.^[48]^ Hence, healthy diet and lifestyle management are highly necessary in people with a high-risk for MetS and cardiometabolic diseases, such as abdominal obesity, elevated BP, glucose levels, and/or reduced HDL-C levels and TG levels.

Meanwhile, a high carbohydrate consumption and total energy intake were observed in healthy participants. Previously, we found no associations between daily intake of carbohydrate-rich foods, such as white rice or mixed rice, while high intakes of wheat (odds ratio (OR): 2.149, 95% CI: 1.134–4.071) and meat (OR: 1.932, 95% CI: 1.066–3.500) were found to be highly associated with the risk of sarcopenic obesity in rural Korean elderly adults with cardiometabolic multi-morbidity.^[49]^ However, it needs to be carefully considered that repeated overconsumption of carbohydrates was related to elevated TG and blood glucose levels with reduced HDL-C levels.^[50,51]^ In particular, elevated BMI is regarded as the predominant risk factor for insulin metabolism and related cardiometabolic disorders, such as insulin resistance and MetS.^[29]^ Therefore, the proper consumption of carbohydrates and energy intake is required in people with healthy status.

### 4.3. Strength and limitations

We used well-validated population-based cohort data to analyze the results. Our results also enabled the observation of MetS and multiple cardiometabolic risk factors based on a number of lifestyle factors. Nevertheless, our study has some limitations. As an observational study, there is a possibility of induced selection bias due to residual and unmeasured confounders. Second, we used <3 years of follow-up data and mostly females (72.4%) therefore, these results may not be generalizable and should be interpreted with caution.

## 5. Conclusions

In conclusion, lifestyle factors and dietary sources were strongly associated with the incidence of abdominal obesity in Korean adults. Moreover, high intakes of vitamin C, fiber and vegetables were negatively associated with the incidence of abdominal obesity. Therefore, screening for enhanced primary care, which focuses on a healthy lifestyle and diet, and great insight with integrative perspectives across public health, strategy, and community are required to improve individual cardiometabolic health.

## Author contributions

**Conceptualization:** Jieun Kim.

**Data curation:** Kyoungsik Jeong.

**Formal analysis:** Jieun Kim.

**Funding acquisition:** Siwoo Lee.

**Methodology:** Jieun Kim.

**Project administration:** Siwoo Lee.

**Supervision:** Younghwa Baek.

**Visualization:** Jieun Kim.

**Writing – original draft:** Jieun Kim.

**Writing – review & editing:** Jieun Kim, Younghwa Baek.

## Supplementary Material


