# Supplementary material for: The association of incident abdominal obesity with diet and unfavorable lifestyle factors in Korean adults: A prospective cohort study
Source: Medicine (Baltimore). 2026 Jan 2;105(1):e46422. doi: 10.1097/MD.0000000000046422 (PMC12778158; doi:10.1097/MD.0000000000046422)
Supplement: Supplementary file 1 [file medi-105-e46422-s001.pdf]

A. MetS

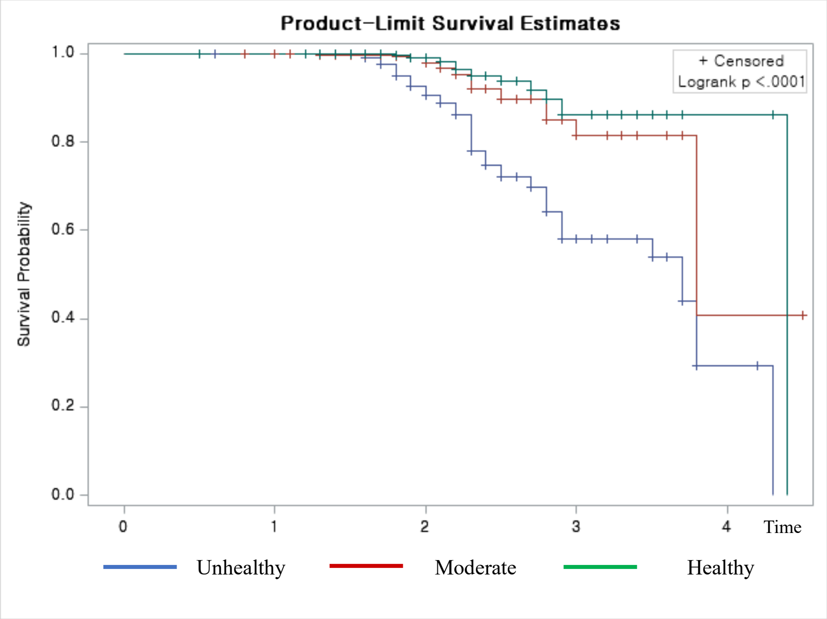

B. Abdominal obesity

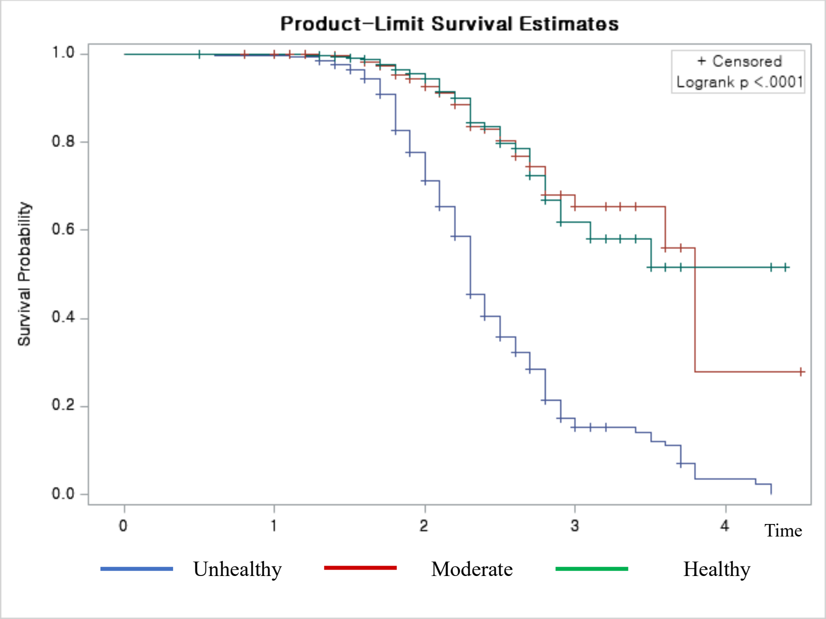

Supplementary 1. Combined lifestyle factors with A) MetS and B) abdominal obesity risk by illustrating Kaplan-Meier curves

Total lifestyle scores were created based on the six lifestyle behaviors (ranging from 0 to 5 factors) and classified into three groups: unhealthy (0-1 factors), moderate (2-3 factors), and healthy (4-5 factors).
